# Transcatheter Occlusion of a Patent Ductus Arteriosus for a Symptomatic Left-to-Right Shunt

**DOI:** 10.7759/cureus.24733

**Published:** 2022-05-04

**Authors:** Papus Keita, Frank Han, Nicholas J Peterman, Sandor Toledo, Anthony Munaco

**Affiliations:** 1 Surgery, Carle Foundation Hospital, Urbana, USA; 2 Pediatric Cardiology, University of Illinois Hospital, Chicago, USA; 3 Medicine, Carle Foundation Hospital, Urbana, USA; 4 Surgery, Lehigh Valley Hospital, Allentown, USA; 5 Surgery, University of Illinois Hospital, Chicago, USA

**Keywords:** pulmonary hypertension, shunt reversal, transcatheter occlusion, patent ductus arteriosus, congenital diaphragmatic hernia

## Abstract

Congenital diaphragmatic hernias (CDH) can induce life-threatening pulmonary hypertension and right heart failure. The patent ductus arteriosus (PDA) is often maintained in CDH to allow for decompression into the systemic circulation. However, if the PDA becomes hemodynamically significant, PDA closure may be indicated. Traditional methods rely on pharmacological closure. In this report, we document a rare transcatheter closure of a hemodynamically significant PDA.

## Introduction

Congenital diaphragmatic hernia (CDH) refers to the complete or partial failure of the diaphragm to form, which can lead to herniation of abdominal content within the thoracic cavity. Subsequently, underdevelopment of the ipsilateral lung, increased pulmonary vascular resistance, pulmonary hypertension, and right heart failure may occur [1]. Mortality rates vary depending on patient factors but are generally estimated to range from 10 to 35% [2]. One of the normal congenital variants, the ductus arteriosus, can serve as a pop-off valve for the elevated pulmonary pressure, allowing for a beneficial pulmonary-to-systemic shunting of blood and the subsequent decompression of the right heart. Therefore, part of the management of CDH is often to maintain a patent ductus arteriosus (PDA) and reduce right heart strain. However, as the treatment of CDH progresses and pulmonary hypertension is reduced, an initially beneficial PDA may reverse its direction of flow and lead to left heart strain [3]. If this shunt results in clinical symptoms, it is termed hemodynamically significant PDA (hsPDA). In this scenario, closure may be indicated to eliminate the hemodynamically significant shunt. Methods for closure have traditionally employed medications such as non-steroidal anti-inflammatory drugs or open surgical ligation. In this report, we describe the transcatheter closure of a hsPDA after congenital diaphragmatic hernia repair.

## Case presentation

**Figure 1:**
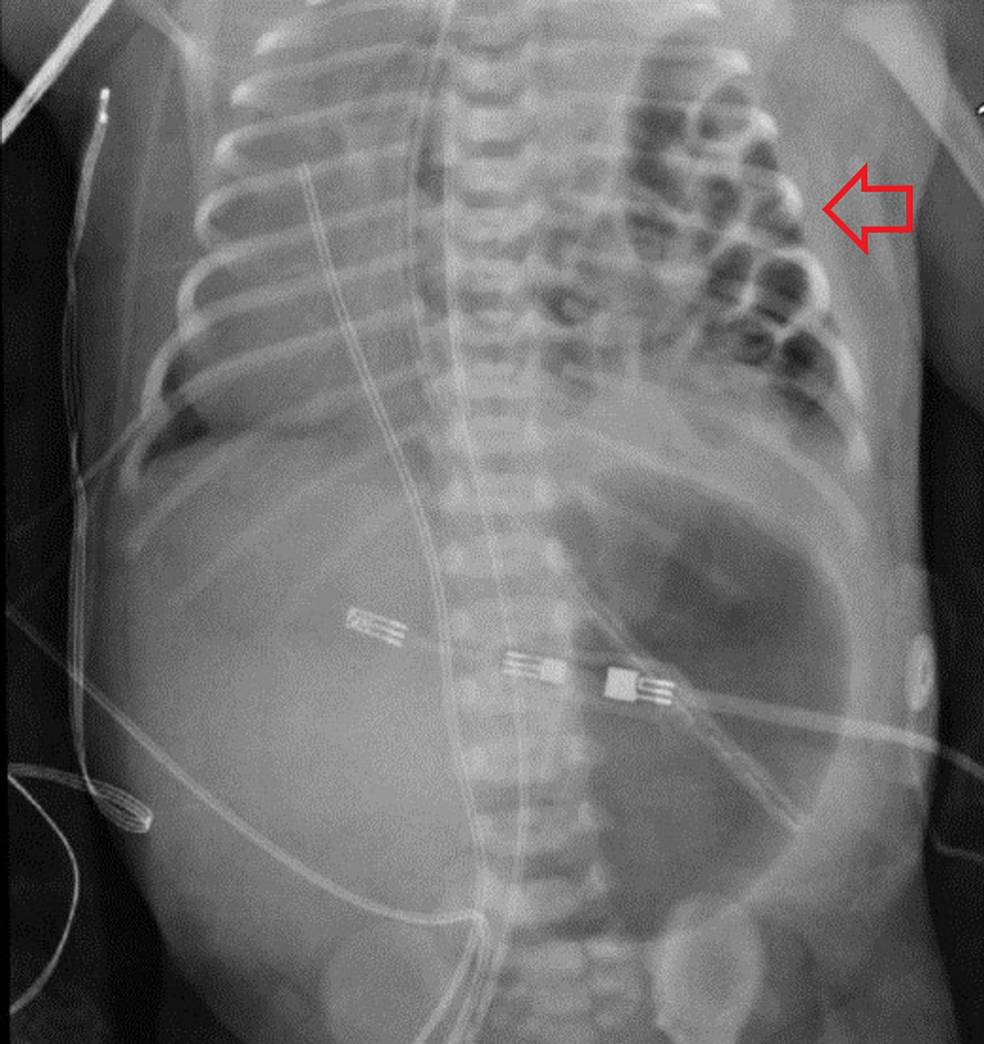
Chest X-ray of the congenital diaphragmatic hernia (arrow)

The patient was a 39-week-old female with a prenatal diagnosis of CDH who had been born via cesarean section for breech presentation. The initial perinatal evaluation was notable for APGAR scores of 5 and 8 at the one- and five-minute marks, respectively. The patient was intubated for respiratory distress. Initial chemistry and hematology were unremarkable. Post-intubation, a chest X-ray confirmed the prenatal diagnosis of CDH (Figure 1), and an echocardiogram showed no congenital heart disease. A repeat echocardiogram was performed on day five and it demonstrated a large PDA with right-to-left shunting and a tiny ventral septal defect (VSD). Pulmonary hypertension was also demonstrated at this time, and sildenafil 1 mg/kg/day was started.

On day one after intubation, pre- and post-ductal saturations remained 100% and 97%, respectively, on 68% FiO_2_, which was serially decreased to 40% FiO_2_ over the next 12 days. On day 13, CDH was repaired without complications, and the FiO_2_ was decreased to 21% by postoperative day three before extubation to non-invasive positive pressure ventilation (NIPPV) on day five. Over the following two days, the patient developed intermittent tachycardia, which prompted a septic workup from which blood cultures grew gram-positive cocci in clusters. The patient was appropriately treated with antibiotics; however, cardiology was consulted after tachycardia persisted. The cardiology team wished to balance the need to treat the pulmonary hypertension with the need to avoid overloading the left atrium and left ventricle. Thus, they began planning for PDA closure.

Enteral feeds were incrementally increased until postoperative day 17, when the patient was diagnosed with enterocolitis with pneumatosis intestinalis on imaging. Ultimately, she had a course of antibiotics with a satisfactory resolution of her abdominal pathology, although persistent tachycardia with associated tachypnea remained. Her respiratory rate soon climbed to the 80s, and she was unable to tolerate oral feeds. Over the next several weeks, aspiration and sepsis were ruled out. On postoperative day 45, an echocardiogram demonstrated a patent foramen ovale (PFO), a hsPDA with flow reversal from right-to-left to left-to-right, and a severely dilated left atrium. The dilated left atrium was attributed to the large venous return from the PDA and its continued patency due to sildenafil. Supportive care was attempted with diuresis and dose reduction of sildenafil. The patient’s tachypnea persisted and a hsPDA was reconfirmed on the echocardiogram, leading to a cardiac catheterization five days later for endovascular closure of the PDA. The PDA was 5.8 mm, and the pulmonary-to-systemic flow ratio was 4:1. It was occluded with an 8-mm vascular plug. Post-procedurally, the patient’s diuresis and sildenafil were slowly weaned off. Echocardiogram confirmed no obstruction to aortic or pulmonary artery flow and no residual shunt. The tachypnea resolved, and she was subsequently able to tolerate enteral feeds. She was discharged 11 days after PDA occlusion.

## Discussion

This case represents an unusual presentation of an early symptomatic PDA in the context of CDH repair. As described in the literature, pulmonary overcirculation can be the etiology of early respiratory symptoms [4]. In our case, there were two challenges. The first was the high index of suspicion that was needed to recognize that the patient’s pulmonary symptoms were secondary to her PDA. Naturally, once the echocardiogram was obtained and the shunt was quantified, it became part of the differential diagnosis. Once the symptoms were attributed to the PDA, we encountered the second challenge, which was the still-ongoing controversy on the best timing of PDA closure [5].

One of the major management challenges in patients with CDH is the concomitant pulmonary hypertension. In normal perinatal physiology, pulmonary resistance significantly drops shortly after birth, creating a differential gradient between the aorta and pulmonary artery that favors shunting away from the systemic circulation. In other words, since the systemic circulation has higher pressures than the pulmonary circulation, a left-to-right shunt ensues until the PDA closes either spontaneously or iatrogenically. In the context of CDH, the pulmonary artery pressure remains supra-systemic. When combined with a PDA, the high afterload can lead to left ventricular strain and failure. In this case, a PDA serves as a pop-off valve into the systemic circulation, thereby lessening the right ventricular afterload.

Historically, PDAs were thought to correlate with increased morbidity, especially in infants with associated defects such as CDH [5-7]. However, this has been a matter of debate of late [6,7]. Ultimately, the relevance of the PDA mostly lies in its association with other defects as well as its hemodynamic significance. Indications for PDA closure remain controversial as its effect on patient outcomes can be sometimes questionable. With regard to the hemodynamic significance, the pertinent echocardiographic signs of PDAs have been previously elucidated by Arlettaz [8]. In our report, the echocardiogram demonstrated a 4:1 pulmonary-to-systemic flow ratio, which is consistent with pulmonary overcirculation [8]. Furthermore, the unexplained tachypnea was likely a symptomatic manifestation of the patient's defect. Given the echocardiogram and clinical presentation, we believe that most experts would agree that a PDA closure was indicated. Ultimately, the temporal association with the patient’s symptom resolution and clinical improvement favor closure as the definitive treatment in our case.

Another decision point in our algorithm was the method of closure. In 2020, Su et al. recapitulated the various therapeutic options for the closure of PDAs [9]. The current standard of care is either pharmacologic closure or surgical ligation, which involves a thoracotomy. However, in recent years, cardiologists have moved towards transcatheter closure in patients with suitable anatomy as initial restrictions on preterm infants have been overcome with the development of smaller profile devices [10]. In select patients, this approach has been shown to have superior rates of technical success and reduced adverse outcomes compared to pharmacologic closure and thoracotomy [11]. To our knowledge, this report represents the first application of the transcatheter PDA closure after CDH repair. While pharmacologic and surgical closure were options, in our case, we felt that transcatheter closure was the most efficacious and least morbid approach to rapid improvement of hemodynamics as well as symptomatic relief given the failure of supportive treatment and the 4:1 pulmonary-to-systemic flow ratio.

## Conclusions

CDH is a rare diagnosis, and it is generally associated with a PDA. Because CDH causes pulmonary hypertension, the PDA is often maintained to reduce right ventricular strain. However, if the PDA becomes hemodynamically significant, PDA closure may be indicated to prevent potential morbidity. A transcatheter approach may be a potential option in select patients.
